# A systematic review of clinical trials affecting anxiety, stress and fear of childbirth in expectant fathers

**DOI:** 10.1002/nop2.681

**Published:** 2020-11-18

**Authors:** Seyedeh Fatemeh Ghaffari, Forouzan Elyasi, Seyed Nouraddin Mousavinasab, Zohreh Shahhosseini

**Affiliations:** ^1^ Student Research committee Mazandaran University of Medical Sciences Sari Iran; ^2^ Psychiatry and Behavioral Sciences Research Center Sexual and Reproductive Health Research Center Addiction Institute Mazandaran University of Medical Sciences Sari Iran; ^3^ Health Sciences Research Center Mazandaran University of Medical Sciences Sari Iran; ^4^ Sexual and Reproductive Health Research Center Mazandaran University of Medical Sciences Sari Iran

**Keywords:** anxiety, childbirth, fathers, fear, nurses, nursing, stress, trials

## Abstract

**Aim:**

To investigate clinical trials affecting anxiety, stress and fear of childbirth in fathers.

**Design:**

A systematic literature search was conducted based on Cochrane Collaboration statement recommendation and Preferred Reporting Items for Systematic reviews and Meta‐Analyses checklist.

**Methods:**

With assistance of Medical Subject Headings, keywords were employed to search for relevant trials. Articles published between November 2000–November 2019 were searched in five electronic databases including PubMed, Web of Science, Google Scholar, Scopus and Cochrane as well as Iranian databases. The risk of bias was assessed by Cochrane Risk of Bias Scale.

**Results:**

A total of eight studies met the inclusion criteria. Interventions were classified into four categories including pre‐natal education, music therapy, massage therapy and relaxation training. The results showed that there is no evidence of a best intervention, but it showed that non‐pharmacological interventions can decrease anxiety, stress and fear of childbirth and increase the positive experience of childbirth in the expectant fathers.

## 1 INTRODUCTION

Although pregnancy and childbirth is a natural life event, but it can lead to major changes that can be stressful (Ganapathy, 2015). Perceived fear of childbirth and the ways of confronting with it are different in the expectant fathers (Dellmann, 2004). Evidence suggests that expecting fathers will experience emotions such as anxiety, stress and fear during pregnancy and childbirth (Ganapathy, 2015; Meleis et al., 2000; Mercer et al., 1988; Schumacher et al., 2008). These feelings are experienced by 13%–80% of first‐time fathers (Labrague & McEnroe‐Petitte, 2016) and to some extent prepare them for childbirth (Ganapathy, 2015; Meleis et al., 2000; Mercer et al., 1988; Schumacher et al., 2008), but an excessive level can be physically and emotionally debilitating (Alessandra & Roberta, 2013). The underlying causes for these feelings are partner pain, feelings of helplessness, lack of knowledge, fear of instrumental delivery and risks endangering the health of mother and her foetus (Eriksson et al., 2006; Hanson et al., 2009).

In clinical practice, fear levels are commonly divided into "low fear," "moderate fear," "severe fear" and "phobic fear" (Larsson, 2017). Approximately 13% of fathers experience severe fear of childbirth (Hildingsson et al., 2014). These feelings are most evident during pregnancy as labour approaches, so it seems to be the best time for effective interventions in the third trimester of pregnancy (Escott et al., 2009; Johnson & Slade, 2002). If these feelings are ignored, they may have consequences such as excessive anxiety, stress and fear of childbirth that increase the risk of developing physical and psychological disorders, hypertension, depression and post‐traumatic stress disorder (Bergström et al., 2013; Hosseini et al., 2018). They may affect children's attachment patterns, emotional and cognitive development of the children, couple relationships, father's ability to support their spouses and an unpleasant experience of pregnancy and childbirth (Bergström et al., 2013; Ganapathy, 2015; Hanson et al., 2009).

## 2 BACKGROUND

Although single studies with controversial results have been conducted on the management of anxiety, stress and fear of childbirth in the expectant fathers (Bergström et al., 2009; Bergström et al., 2013; Charandabi et al., 2017; Labrague & McEnroe‐Petitte, 2016; Latifses et al., 2005; Li et al., 2009; Mihelic et al., 2018; Wöckel et al., 2007), no systematic review has reported the effectiveness of clinical trials to reduce these problems in this group. In this way, it showed that fathers’ education in pre‐natal period can lead to a positive childbirth experience and reduces their fear (Bergström et al., 2013; Li et al., 2009), although another study failed to approve the effectiveness of pre‐natal educational programme on couples' stress during pregnancy (Bergström et al., 2009).

According to the International Conference on Population and Development agenda, the men's involvement in all aspects of sexual and reproductive health matters including pregnancy and childbirth is an important issue to meet safe motherhood (Jamali et al., 2018). So pay attention to the expectant fathers' mental health to support their spouses during pregnancy is a priority. It is important to understand which interventions will most effectively advance expectant fathers' emotional conflicts; therefore, this systematic review was conducted to assess the clinical trials affecting anxiety, stress and fear of childbirth in the expectant fathers.

### 2.1 Research questions

- Which clinical trials will most effectively advance the expectant fathers' anxiety, stress and fear of childbirth?
- How clinical trials are effective in reducing anxiety, stress and fear of childbirth in the expectant fathers?

## 3 METHODS

The steps taken to conduct this study were as follow (Emami‐Sahebi et al., 2018; Hosseini et al., 2018):

### 3.1 Study design

A systematic literature search was performed based on Cochrane Collaboration statement recommendation and Preferred Reporting Items for Systematic reviews and Meta‐Analyses checklist (PRISMA) (Higgins et al., 2011; Moher et al., 2011).

### 3.2 Formulating the research question

As mentioned above, the research question was posed.

### 3.3 Extracting keywords

The following keywords and their equivalents in the Persian language were used to retrieved articles: ["Anxiety" and "Stress" and "Fear" OR "Tokophobia"] AND ["Childbirth" OR "Delivery" OR "Parturition" OR "Birth"] AND ["Pregnancy" OR " Gestation"] AND ["Father" OR "Men" OR "Couple" OR "Paternal"] AND ["Intervention" OR "Method" OR "Management" OR "Therapy" OR "Education" OR "Counseling" OR "Hypnosis" OR "Music therapy" OR "Relaxation" OR "Massage therapy"] AND ["Randomized Clinical trial"].

### 3.4 Searching databases

Articles published between November 2000–November 2019 were searched in electronic databases, such as PubMed, Web of Science, Google Scholar, Scopus and Cochrane as well as the Iranian databases including Magiran, IranMedex and Scientific Information Database. A manual search of references linked to the articles of concern was also conducted. Final search was made on 20 November 2019.

### 3.5 Extracting articles on the basis of the selection criteria

Initially, through an advanced systematic search the articles were extracted. After omitting repeated cases, the remaining irrelevant articles were eliminated by reviewing title, abstract and the main text. Article selection was done independently by two individual co‐workers (Z.Sh and SF.Gh). In the case of a disagreement, selection was done by a third author (F.E). The PRISMA checklist guided the reporting process (Moher et al., 2011; Moher et al., 2009).

### 3.6 Inclusion and exclusion criteria

In this review, clinical trials published in the English and the Persian languages addressing the effects of interventions on anxiety, stress and fear of childbirth in the expectant fathers were included. In cases where the full text of the article was not available, the abstract was used. If the abstract did not provide sufficient information, the article was excluded from the study.

### 3.7 Quality assessment

Included articles were further assessed using Cochrane Risk of Bias Scale which is a recommended tool to assess the risk of bias in the interventional studies. The scale measures biases in case of selection, performance, attrition, reporting and other bias and classifying studies as "low‐risk," "high‐risk" and "unclear" (Higgins et al., 2011).

### 3.8 Data retrieval, classification and reporting

Abstracts and main texts of the eligible articles were carefully studied for the retrieval of required data. Finally, collected data were classified and formally presented.

## 4 RESULTS

### 4.1 Search results

**FIGURE 1:**
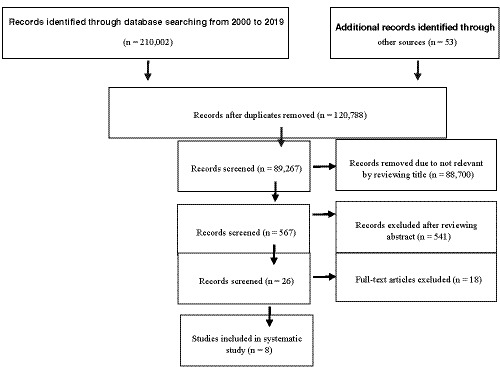
Study selection process

Initially, a total of 210,055 articles were retrieved. Duplicates articles were removed by Endnote software, and then, the articles were reviewed by two authors and the irrelevant articles were deleted. In the next step, 567 papers and full texts of 26 papers were evaluated and eight studies were singled‐out in the final selection phase (Figure 1). Out of those, two dealt with fear of childbirth, four were dedicated to childbirth‐related anxiety, one addressed childbirth‐related stress and one concurrently investigated childbirth‐related anxiety and stress. General description and methodology of the selected literature are described in Table 1.

**TABLE 1:** Characteristics of included studies

Results	Time of outcome measurement	Outcome measurement	Kind of intervention	Participant	country	Date of publication	First author
Massage therapy significantly reduces anxiety compared with the control group (MD = 4.05, Std. error = 0.77, *p* = .001) and the relaxation group (MD = 4.05, Std. error = 0.77, *p* = .001).	End of the 5‐week study period	STAI	IG: Two times a week for 20 min each time Intervention: 1) Massage therapy 2) Relaxation	175 adult years' expectant fathers with their partner in 24–32 gestational weeks There were at least 54 participant in each group.	USA	2005	Latifses, V.
A striking difference was seen in the verification of the fears (before intervention: father with fear of childbirth in CG = 26, father with fear of childbirth in IG = 34. After delivery: father with fear of childbirth in CG = 20, father with fear of childbirth in IG = 10, p = not reported).	three months after the birth	Researcher‐made questionnaire	IG: 12‐hr classes for couples and an hour of special training for men that began around four months before the estimated delivery date Intervention: Discussed theoretical facts about pregnancy, birth and the weeks following childbirth	100 couples were having their first child with aged 28–34 years old and partners aged 28–32 years old with 6 weeks of pregnancy CG:48 IG:52	German	2007	Woeckel, A.
Significant reduction of anxiety (*F* = 3.38, *p* = .001).	Two hours after delivery	STAI for Adults	IG: 4‐hr classes with 3–4 couples in the third trimester of pregnancy Intervention: Birth Education Programme	87 first time expectant fathers that their partners were at 34 to 36 weeks’ gestation, age was 20 years or more and were willing to attend the delivery from the beginning to the end CG:42 IG:45	Taiwan	2009	Tzu‐Li, H
No statistically significant differences in the parental stress (RR = 0.1, 95% CI: 0.0 to 0.1, *p* = .4)	3 months after birth	SPSQ	IG: four 2‐hr sessions during pregnancy and one follow‐up session within 10 weeks after delivery Intervention: Antenatal education model	1,087 nulliparous women and 1,064 of their partners CG: 106 groups IG: 101 groups	Sweden	2009	Bergstrom, M
Significant reduction of fear of childbirth (Adjusted OR = 0.30, 95% CI: 0.10–0.95, p = not reported).	mid‐pregnancy 3 months after birth	W‐DEQ A W‐DEQ B	IG: Four two‐hour sessions during pregnancy and one follow‐up session within 10 weeks after delivery Intervention: Psychoprophylaxis model	83 men that came with their partner in 19 gestational weeks to receive antenatal care and suffered from fear of childbirth CG:44 IG:39	Sweden	2013	Bergstrom, M
Significant reduction of anxiety (95% CI: −7.07 to −5.53, *t* = 16.23, *p* < .05).	30 min after intervention	STAI	IG: Exposed to music intervention for 30 min when their partner/wife was admitted into the delivery room. Intervention: Music Therapy	98 first‐time fathers with aged 18 years or older CG:48 IG:50	Philippines	2016	Labrague, L.
Significant reduction of state anxiety and trait anxiety (8 weeks after intervention: state anxiety AD = −5.7, 95% CI: −8.6 to −2.9 *p* < .001, trait anxiety AD = −5.0, 95% CI: −7.8 to −2.2, *p* = .001; 6 weeks after childbirth: state anxiety AD = −7.5, 95% CI: −11.6 to −3.4, *p* < .001, trait anxiety AD = −8.3, 95% CI: −12.2 to −4.4, *p* < .001).	8 weeks after the intervention and 6 weeks after the childbirth	STAI	IG: Two weekly training sessions (lasting 60–90 min between 24–28 weeks) Intervention: Life style‐based education	125 spouses of pregnant women with gestational ages of 24–28 weeks CG: 63 IG: 62	Iran	2017	Mohammad‑Alizadeh Charandabi, S.
No significant intervention effects for Baby Triple P were found at either post‐ or follow‐up assessments (DASS‐Anxiety: T1‐T2 95% CI: −0.50 to 0.23, T1‐T3 95% CI: −0.49 to 0.25, *p* = .653; DASS‐stress: T1‐T2 95% CI: −0.62 to 0.12, T1‐T3 95% CI: −0.46 to 0.28, *p* = .690).	pregnancy, 10 weeks’ postbirth and 6 months’ postbirth	DASS−21	IG: four 2‐hr group sessions conducted during pregnancy between the 20th–40th weeks of gestation, followed by four individual 30‐min telephone sessions conducted postnatally once the baby was 6 weeks old. Intervention: Baby Triple P	112 fathers expecting their first baby CG: 55 IG: 57	Australia	2018	Mihelic, M.

Abbreviations: AD, Adjusted Difference; CG, Control Group; CI, Confidence Interval; IG = Intervention Group; MD, Mean Difference; F, *F*‐test; OR, Odds Ratio; *p*, *p*‐value; RR, Relative Risks; *t*, *t* test; T1, baseline; T2, post‐intervention; T3, 6‐month follow‐up.

### 4.2 Review of studies

#### 4.2.1 Participants

Participants were fathers whose spouses were pregnant. Among the studies that investigated the fear of childbirth, the maternal gestational age at the time of the study was 19 weeks (Bergström et al., 2013) and 6 weeks (Wöckel et al., 2007). In both studies, mothers were nulliparous. In the study conducted by Bergström et al. (2013), only fathers who reported severe fear of childbirth (based on the Wijma questionnaire that was standardized for Swedish fathers) were recruited. In the study conducted by Wöckel et al. (2007), the severity of childbirth‐related fear was not considered as an inclusion criteria. It was compared in the intervention group versus the control group at the baseline and at the postintervention phase via a researcher‐made questionnaire.

Among the studies investigating anxiety in fathers, the mothers were at 2nd trimester (Charandabi et al., 2017; Latifses et al., 2005; Mihelic et al., 2018) or 3rd trimester of pregnancy (Labrague & McEnroe‐Petitte, 2016; Li et al., 2009). They were nulliparous in three of the studies (Labrague & McEnroe‐Petitte, 2016; Li et al., 2009; Mihelic et al., 2018). In the two other studies, mothers were primigravida or multigravida. In the retrieved articles, anxiety [measured by State‐Trait Anxiety Inventory (STAI) or Depression Anxiety Stress Scale‐21(DASS‐21)] was compared in the intervention group versus the control group at pre‐intervention and postintervention periods (Charandabi et al., 2017; Labrague & McEnroe‐Petitte, 2016; Latifses et al., 2005; Li et al., 2009; Mihelic et al., 2018). Of the two studies investigated childbirth‐related stress in fathers, maternal gestational age was 19 weeks (Bergstrom et al., 2009) and 20–35 weeks (Mihelic et al., 2018) at the time of inclusion. In these studies, all mothers were nulliparous that their stress and anxiety were measured by Swedish Parenthood Stress Questionnaire or DASS‐21 (Bergström et al., 2009; Mihelic et al., 2018).

#### 4.2.2 Interventions

##### Prenatal education

Bergström et al. (2013) investigated the effects of pre‐natal education on fathers. Interventions entailed four two‐hour sessions provided in the 3rd trimester of pregnancy and a follow‐up session within the first 10 weeks of postnatal period. In each session, fathers have been educated to act as labour coaches. Also instructions about how to give massage, coach psycho‐prophylactic breathing, encourage relaxation and give emotional support to the mothers were included. Couples were encouraged to practice at home between each session and discuss mental strategies aimed to fear of childbirth reduction.

Woeckel et al. (2007) investigated the childbirth fear in the fathers in a study titled "Getting ready for birth?". Interventions were offered in the form of a 12‐hr education course for couples in both the intervention and the control groups plus an additional one‐hour exclusive session specifically designed for the invention group. Courses began at an estimated time of 4 months and were implemented on a single‐session/week schedule. Theoretical instructions were offered for fathers about natural childbirth, postnatal period, advantages of breast feeding, ways to support spouses including breathing and massage techniques. Issues related to childbirth fear and stress‐triggered physical and psychological responses were also discussed.

Li et al. (2009) investigated the effects of childbirth education programmes on fathers' anxiety. Intervention was implemented in 4‐hr sessions organized in the 3rd trimester with the participation of 3–4 couples. The content of the educational sessions included a. encouraging fathers to share their feelings and anxieties, as well as positive evaluation of their presence during the labour process; b. teaching fathers to better understanding of his partner's physical and psychological changes in the childbirth process, how to help lessen the discomfort of labour pain, offer emotional support and provide physical contact between parents and the baby after childbirth; c. discussing with the expectant fathers about how he could relax and take care of himself; d. sharing personal experiences and information in small groups, watching videos, as well as visiting the labour and delivery rooms. At the end of the educational session, a booklet and a CD were given to the couples to practice at home.

*Mohammadalizadeh* Charandabi et al. (2017) measured the effect of a life style‐based education on the fathers' anxiety and depression during pregnancy and postpartum. Intervention was offered by a male psychologist in two 60‐ to 90‐min sessions per week for a group 5–15 participants, during 24–28 weeks of pregnancy. Educational content included about healthy sleep and diet, physical exercise, self‐recognition and sexual issues.

Bergstrom et al. (2009) investigated the effect of a normal delivery preparation programme on the parenting stress. Intervention was set in four two‐hour sessions delivered during the 3rd trimester. Instructions provided information about childbirth process, non‐pharmacological pain relief, breast feeding, partner's role as a coach during labour, breathing exercises, as well as massaging and relaxation techniques. Psycho‐prophylaxis education was given between sessions, and homework booklet was distributed among participants.

Mihelic et al. (2018) investigated the effect of a perinatal parenting intervention on fathers' anxiety and stress who expecting their first baby. This intervention as named Baby Triple P was on over three time points: pregnancy, 10 weeks after birth, and 6 months after birth. It was provided in form of group as well as phone call counselling sessions about positive parenting, responding to child, individual survival skills and partner support. Individual coping plans were requested as homework and issues concerning undesirable thoughts, communication skills, social support, emotional expression and task sharing were discussed.

##### Music therapy

Labrague & McEnroe‐Petitte (2016) investigated the effects of music therapy on the first‐time expectant fathers' anxiety. The music intervention took place in the fathers' waiting room and started when their partner/wife was admitted into the delivery room. During this period, fathers listened to music (through headphone) for 30 min. Selected music included classical (Claude Debussy, Adagio for Strings, Pachelbel's Canon), country (Teardrops on My Guitar, Lovestory) and the sound of nature.

##### Massage therapy

Latifses et al. (2005) studied how massage therapy given to mothers by their spouses affected anxiety in fathers. Required training was given to fathers by a massage therapist before programme implementation. The fathers massaged their pregnant spouses twice a week.

##### Relaxation

Latifses et al. (2005) investigated the effect of relaxation on reducing anxiety in fathers. Relaxation was taught to couples in weeks 24–32 of pregnancy and at the end of the course, the participants were given a relaxation training audio recording. Couples were required to practice the exercises twice a week (for 20 min each time).

### 4.3 Outcomes and measurements

Outcome of two studies was on fear of childbirth (Bergström et al., 2013; Wöckel et al., 2007), four studies on anxiety (Charandabi et al., 2017; Labrague & McEnroe‐Petitte, 2016; Latifses et al., 2005; Li et al., 2009), a single study on stress (Bergström et al., 2009) and another single study on both stress and anxiety (Mihelic et al., 2018). In one of the studies, fear of childbirth was assessed by Wijma Delivery Expectancy/Experience Questionnaire (W‐DEQ A/B) which is a standard tool for measuring childbirth fear in women (Bergström et al., 2013; Wijma et al., 1998). To apply the scale to fathers, Bergström et al. (2013) evaluate its psychometric properties among Swedish fathers. They found that fear of childbirth decreased in the antenatal education group compared with the standard care group [Adjusted Odds Ratio (AOR) = 0.30, 95% Confidence Interval (95% CI): 0.10–0.95]. Woeckel et al. (2007) used a culture‐based tool in their investigation to measure fear of childbirth in fathers. The tool is based on Lukeach standard childbirth fear questionnaire and is used for evaluating childbirth experience, information source and spouse assistance during labour as well. They found that education sessions and discussions about pregnancy and childbirth reduced the fear of childbirth in 70% of fathers in the intervention group. The most of the fathers in this group (96%) reported a positive experience compared with 72% of the control group (*p* = .0001).

In most studies, STAI was used to measure anxiety (Charandabi et al., 2017; Labrague & McEnroe‐Petitte, 2016; Latifses et al., 2005; Li et al., 2009). However, in the study conducted by Mihelic et al. (2018), depression, anxiety and stress were measured by means of DASS‐21. Mihelic et al. (2018), additionally evaluated postnatal depression in fathers using Edinburgh Postnatal Depression Scale (EPDS), personal happiness using Oxford Happiness Questionnaire (OHQ), parenting confidence and sense of competency using Maternal Self Report Inventory (MSRI), couple relationship using the contents of several questionnaires, father's feeling about infant investigated by Maternal Infant Responsiveness Instrument (MIRI) and father's attachment to infant evaluated by Postpartum Bonding Instrument (PBI). They showed that their intervention (Baby Triple P) had no significant effect on anxiety (AOR = −0.13, 95% CI: −0.50 to 0.23, *p* = .653), stress (AOR = −0.25, 95% CI: −0.62 to 0.12, *p* = .690), depression (AOR = 0.12, 95% CI: −0.25 to 0.49, *p* = .618), personal happiness (AOR = 0.34, 95% CI: −0.03 to 0.71, *p* = .350), confidence (AOR = −0.24, 95% CI: −0.61 to 0.13, *p* = .656) and couple relationship (AOR = −0.23, 95% CI: −0.60 to 0.14, *p* = .135).

In the study conducted by Li et al. (2009), in addition to fathers' anxiety, childbirth expectations questionnaire developed by Gupton et al. (1991) was employed to evaluate fathers' expectation about the childbirth experience and the crucial cares under different circumstances. Accordingly, after analysis of covariance to justify covariate variables including education level, sources of childbirth information, attendance at Lamaze childbirth classes and childbirth expectations they reported that a birth education programme based on the self‐efficacy theory can reduce an expectant fathers' anxiety (*F* = 3.38, *p* = .001). They also found that all expectant fathers' partners were the most popular source of the information about childbirth (57.8% and 69.0% for the experimental and the control groups, respectively).

Labrague et al. (2016), in addition to anxiety, assessed the satisfaction with the childbirth experience by a Visual Analogue Scale (VAS) at 2 hr after childbirth. Results revealed that the first‐time fathers in the experimental group (that received music therapy) had lower anxiety score [Mean Difference (MD) = 16.23, 95% CI: −7.07 to −5.53, *p* < .05] and higher satisfaction scores (MD = 14.53, 95% CI: 2.15–2.83, *p* < .05) than those in the control group. Latifses et al. (2005), besides, evaluation of massage therapy and relaxation on fathers' anxiety, measured by STAI, investigated marital adjustment with the Dyadic Adjustment Scale (DAS). They showed that massage therapy compared with relaxation/ no intervention decreased the fathers' self‐reported anxiety levels (MD massage‐control = 4.05, *p* = .001; MD massage‐relaxation = 4.03, *p* = .001) and improved marital adjustment (MD massage‐control = 5.28, *p* = .022; MD massage‐relaxation = 5.35, *p* = .023). *Mohammadalizadeh* Charandabi et al. (2017) evaluated both anxiety and depression during pregnancy and after childbirth. In their cases, depression evaluation was done by means of EPDS. The results showed a positive effect of the life style‐based education on the pre‐natal and the postnatal depression as follows: Pre‐natal depression: AOR = −1.60, 95% CI: −2.80 to −0.50, *p* = .004; Postnatal depression: AOR = −3.30, 95% CI: −5.00 to −1.50, *p* = .001. Pre‐natal state anxiety: AOR = −5.70, 95% CI: −8.60 to −2.90, *p* < .001; Postnatal state anxiety: AOR = −7.50, 95% CI: −11.60 to −3.40, *p* < .001. Pre‐natal trait anxiety: AOR = −5.00, 95% CI: −7.80 to −2.20, *p* = .001; Postnatal trait anxiety: AOR = −8.30, 95% CI: −12.20 to −4.40, *p* < .001.

In the study carried out by Bergstrom et al. (2009), parental stress was measured using the SPSQ. This questionnaire is based on American questionnaire parenting stress index (Östberg et al., 1997). In this study, childbirth expectation and childbirth experience of the parents were measured separately by means of W‐DEQ A/B. They reported that antenatal education did not make a statistically significant difference in the experience of childbirth [Relative Risks (RR) = −1.60, 95% CI: −0.70 to 4.00, *p* = .1] or parental stress (RR = −0.10, 95% CI: 0.00–0.10, *p* = .4) between groups.

### 4.4 Quality assessment

**FIGURE 2:**
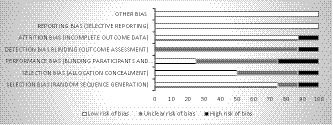
Review authors’ judgments about each risk of bias item as percentages across all included studies

**FIGURE 3:**
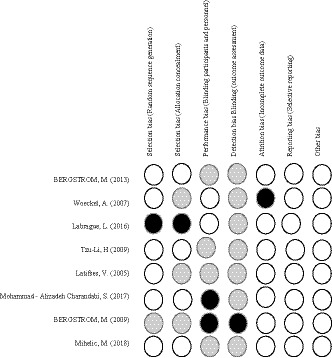
The results of risk of bias evaluation for each included study

Results of quality assessment of the eight clinical trials are presented in Figures 2 and 3. Two reviewers (Z.Sh, SF.Gh.) had good agreement, and any discrepancies were resolved following discussion. Four studies were assessed as low risk of bias (Bergström et al., 2013; Latifses et al., 2005; Li et al., 2009; Mihelic et al., 2018) and four as high risk of bias (Bergström et al., 2009; Charandabi et al., 2017; Labrague & McEnroe‐Petitte, 2016; Wöckel et al., 2007).

## 5 DISCUSSION

The present systematic review focuses on studies that investigated the effects of clinical trials on anxiety, stress and fear of childbirth in the expectant fathers. As literature showed, childbirth can be a tremendously stressful experience for fathers, triggering anxiety and fear of childbirth (Ganapathy, 2015; Labrague & McEnroe‐Petitte, 2016; Meleis et al., 2000; Mercer et al., 1988; Schumacher et al., 2008). Mismanagement of these issues may discourage fathers from expressing these feelings which, in turn, has its adverse consequences (Chandler & Field, 1997; Eriksson et al., 2007).

Our investigation indicates the most intervention implemented to reduce anxiety, stress and fear of childbirth was educational trials (Bergström et al., 2009, 2013; Li et al., 2009), although the results are controversial (Bergström et al., 2009; Hallgreen et al., 1999; Smith, 1999). Given that one of the reasons for poor emotional feelings during pregnancy is the lack of knowledge about pregnancy and childbirth (Hanson et al., 2009; Salehi et al., 2016), so healthcare providers with providing appropriate education can take an important step. These educations could be organized in form of individual or group activities and be taught through classes, reading documents, watching videos, discussing or be Internet‐based (Bergström et al., 2013; Stark & Nursing, 1997). The content of the educational courses could be including information about the natural process of childbirth, possible complications, ways to reduce pain and the causes of fear of childbirth. There should also be an opportunity for fathers to discuss their feelings. These educations elevate confidence in fathers by increasing their knowledge and skills that needed to help them to play a more constructive role in childbirth processes (Li et al., 2009).

Our study showed the beneficial effects of music, massage and relaxation on the expectant fathers' anxiety in a few limited trials (Labrague & McEnroe‐Petitte, 2016; Latifses et al., 2005). It seems music through the brain affects the noradrenaline, cortisol, endorphins and adrenocorticotropic (McCaffrey et al., 2020; Spintge & Droh, 1987). Music intervention programmes can be recognized as a leading non‐invasive cost‐effective approach targeting the rising costs of healthcare services worldwide (Browning, 2001; Labrague & McEnroe‐Petitte, 2016). Also, massage as a one of the most popular complementary and alternative medical treatments for anxiety (Ardianti et al., 2020; Hall et al., 2020) can improve the expectant fathers' anxiety (Latifses et al., 2005). Learning and practicing massage by fathers makes fathers feel closer to their spouses and their foetuses, reduces pre‐natal and postpartum tensions and also increases marital satisfaction and ultimately improves parental relationships. Relaxation is another intervention that creates a feeling of calm by affecting the autonomic nerves. Nonetheless, Latifses et al. (2005) failed to discover any evidences in support of anxiety‐reducing effects of relaxation.

This study can be helpful to obstetricians, midwives, psychologists and psychiatrists in managing anxiety, stress and fear of childbirth in spouses of pregnant women and thus improve the quality of fathers' health and subsequent family health. The strengths of this review include a rigorous literature search, use of a validated methodology and use of two independent reviewers during data evaluation, data extraction and synthesis. There is a possibility of publication bias as only published data were included. Also, some potential limitations need to be acknowledged. Firstly, the methodological quality of the studies varied, which might have affected the results. Given the heterogeneity of the populations enrolled in terms of characteristics and sample sizes, findings should be taken with particular caution. Secondly, differing definitions of anxiety, stress and fear of childbirth in fathers, including different measurement tools and cut‐offs, were used so meta‐analysis could not be performed.

## 6 CONCLUSION

This systematic review cannot provide any evidence to support the superiority of one intervention over another. Hence, verification of the effectiveness of interventions for the management of anxiety, stress and fear of childbirth demands additional probe and contemplation.

## 7 ACKNOWLEDGEMENT

The current review is part of a master's thesis by Seyedeh Fatemeh Ghaffari, a master's student in midwifery counselling. Hereby, we express our gratitude to the Deputy of Research in Mazandaran University of Medical Sciences.

## 8 ETHICAL CODE

IR.MAZUMS..REC.1399.6848

## CONFLICT OF INTERESTS

The authors have no conflicts of interest relevant to this article.

## CONFLICTS OF INTEREST

There are no conflicts of interest.

## AUTHORS’ CONTRIBUTIONS

SF. Ghaffari and Z. Shahhosseini: Designing and conducting the study and manuscript writing; Z. Shahhosseini and F. Elyasi: Editing the final manuscript and providing critical revision; SN. Mousavinasab: Statistical analyses. All authors: Manuscript reading and approval.

## Data Availability

Data will be available upon your request.
